# What About Foods? The Influence of Food Texture on the Safety, Timing, Kinematics, and Efficiency of Pharyngeal Phase Swallowing in Healthy Adults

**DOI:** 10.1044/2025_JSLHR-25-00546

**Published:** 2026-03-26

**Authors:** Pooja Gandhi, Emily Barrett, Renata Mancopes, Vanessa Panes, Melanie Peladeau-Pigeon, Michelle M. Simmons, Catriona M. Steele

**Affiliations:** a Department of Communication Sciences and Disorders, Faculty of Rehabilitation Medicine, University of Alberta, Edmonton, Canada; b Swallowing Rehabilitation Research Laboratory, KITE Research Institute—University Health Network, Toronto, Ontario, Canada; c Department of Speech-Language Pathology, Rehabilitation Sciences Institute, Temerty Faculty of Medicine, University of Toronto, Ontario, Canada; d Canada Research Chair (Tier 1) in Swallowing and Food Oral Processing, Canada Research Chairs Secretariat, Ottawa, Ontario

## Abstract

**Purpose:**

Solid food stimuli (e.g., crackers) are commonly used in videofluoroscopic swallowing studies (VFSS). A variety of additional food textures may be included when exploring the benefit of compensatory strategies. However, interpretation regarding these textures is hindered by a lack of data outlining expected values for measures of swallowing safety, kinematics, timing, and efficiency. We report preliminary data for quantitative VFSS measures with International Dysphagia Diet Standardisation Initiative (IDDSI) food levels MM5 (minced and moist), SB6 (soft-and-bite-sized), and RG7 (regular) in healthy young adults.

**Method:**

VFSS were performed at 30 frames per second in 20 participants (10 men, 10 women; *M*_age_ = 28 years, range: 23–55) who swallowed two boluses each of MM5 (teaspoon), SB6 (cube 1.5 cm^3^), and RG7 (bite) barium stimuli. Blinded duplicate rating identified key frames on the initial swallow of each bolus, from which timing measures were derived relative to hyoid burst (HYB) and end of aggregation (EOA). Safety was scored using the 8-point Penetration–Aspiration Scale. Anatomically normalized pixel-based measures of pharyngeal area at maximum pharyngeal constriction (PhAMPC), upper esophageal sphincter maximum distention (UESMAX), and residue were obtained. Intraclass correlations were calculated for reliability; discrepancies were resolved by consensus. Friedman's tests explored differences by texture. The results were compared to previously reported reference data for teaspoons of EX4/PU4 using one-sample *t* tests.

**Results:**

Timing measures relative to HYB increased across textures: MM5 < SB6 < RG7 and were significantly longer than reported values for EX4/PU4. Timing measures calculated relative to EOA did not differ by texture. UES opening duration was significantly longer for MM5 than SB6, RG7, and EX4/PU4. UESMAX distention was significantly smaller for RG7 and EX4/PU4 than MM5 and SB6. PhAMPC was larger for MM5, SB6, and RG7 than EX4/PU4. Total pharyngeal residue was significantly greater for MM5 and RG7 than for SB6 and EX4/PU4.

**Conclusions:**

These data suggest that variations in some pharyngeal phase parameters should be expected across IDDSI levels EX4/PU4, MM5, SB6, and RG7. Additional research is needed to elucidate interactions of bolus size and cohesiveness with these texture-related differences. Teaspoons of EX4/PU4 stimuli are insufficient to predict swallowing physiology with higher food texture levels. RG7 stimuli are recommended to test pharyngeal constriction and swallowing efficiency. SB6 stimuli can provide additional insights regarding the benefits of altering bolus properties in terms of swallowing efficiency.

**Supplemental Material:**

https://doi.org/10.23641/asha.31592143.

Management of dysphagia typically involves two primary strategies: rehabilitation to restore swallowing function and compensatory techniques to temporarily facilitate improved swallowing safety and/or efficiency. Current clinical practice relies heavily on the compensatory strategy of recommending thickened liquids and texture-modified foods. Thickened liquids are understood to facilitate improved swallowing safety by slowing bolus flow, providing additional time to achieve laryngeal vestibule closure (LVC; Borders & Steele, 2024; Steele et al., 2023). Texture-modified foods are commonly recommended with the goals of reducing the need for chewing and facilitating formation of a cohesive, swallow-ready bolus. Cohesive and moist foods are less likely to fragment during the swallowing process, mitigating the risk of aspiration and choking incidents (Cichero et al., 2013; Mann et al., 2013; Raheem et al., 2021). The International Dysphagia Diet Standardisation Initiative (IDDSI) provides a global framework for standardizing terminology related to texture-modified diets, specifying eight levels of consistency, with drinks spanning Levels 0–4 and foods spanning Levels 3–7 (Cichero et al., 2017, 2020).

Despite wide-spread use, recommendations for diet-texture modification come with some negative consequences. Patients find altered textures unpalatable (Logemann et al., 2008), and studies suggest that the preparation and use of pureed and minced foods in aged care facilities is associated with nutrient depletion and increased risk of malnutrition (Vucea et al., 2017, 2018). Therefore, it is important that diet-texture recommendations be based on comprehensive assessment (Royal College of Speech and Language Therapists, 2024). Evidence regarding both the pros and cons of diet-texture modification can be gathered from instrumental assessments, such as the videofluoroscopic swallowing study (VFSS), also known as the modified barium swallow. During this procedure, boluses of various consistencies are observed traveling through the upper aerodigestive tract. Current recommendations regarding standard VFSS protocols involve a variety of liquid consistencies, but only a single food item (often a cracker smeared with contrast material) is typically used to capture information about swallowing on food textures (Hutcheson et al., 2017, 2022; Leonard et al., 2004; Martin-Harris et al., 2008; Scharitzer et al., 2025; Steele et al., 2021). This reflects the fact that VFSS was never intended to include a full range of table foods (Logemann, 1993). Instead, given the lack of regulatorily approved contrast materials representing different food textures, and concern that exploration of multiple food textures increases radiation exposure, testing of a single food texture has been recommended to interrogate physiologic function, and identify risks of airway invasion, choking, and residue (Steele et al., 2021). Standard approaches to performing VFSS exams also advocate for exploratory testing of selected compensatory strategies when dysfunction is noted on standard consistencies, or, in the case of apparently safe and efficient swallowing on standard tasks, further provocative testing with tasks intended to challenge the system (Jones & Donner, 1989; Logemann, 1993; Scharitzer et al., 2025). In addition to behavioral or positional strategies, this further testing may include a variety of different food stimuli, including items representative of IDDSI levels MM5 (minced and moist), SB6 (soft-and-bite-sized), mixed consistencies, pills, and items reported by the patient to be particularly challenging, all mixed in an off-label fashion with contrast media. Currently, there is a paucity of reference data available to guide the interpretation of videofluoroscopy with these nonstandard food stimuli. Developing such reference information is critical to inform clinical practice, by confirming the generalizability of measures of swallowing function and physiology on standard consistencies (IDDSI Level EX4/PU4 extremely thick liquids/pureed foods or IDDSI Level RG7 regular foods) to other levels on the IDDSI framework.

Prior videofluoroscopic studies of healthy chewing, oral processing, and swallowing with solid foods primarily comprise of the seminal work of Karen Hiiemae and Jeffrey Palmer, who tracked movement of the jaw, hyoid, tongue, and bolus during chewing, oral processing, and swallowing in a small sample of healthy adults (Hiiemae & Palmer, 1999). A key finding from that work was the identification of two distinct stages in food oral processing, known as Stages I and II transport. In Stage I, food is moved to the postcanine teeth by a pull-back tongue movement and chewed for different time intervals depending on initial consistency. In Stage II, portions of chewed food are transported through the faucial isthmus to the oropharynx through a tongue squeeze-back mechanism. Oral food processing may continue during Stage II transport. Several portions of prepared food may be transported as separate aliquots into the valleculae, where a bolus gradually accumulates while cyclic jaw movements and tongue squeeze-back events are ongoing (Hiiemae & Palmer, 1999; Palmer et al., 2007). Hiiemae and Palmer gave this accumulation pattern the name “postfaucial aggregation,” and described it as a normal phenomenon that could last as long as 10 s prior to initiation of the pharyngeal swallow. Bolus aggregation with solid foods contrasts with the pattern that is expected with liquids, namely, bolus transfer without cyclic jaw movement and rapid swallow initiation once the bolus enters the pharynx (Kendall et al., 2000; Molfenter & Steele, 2012; Namasivayam-MacDonald et al., 2018; Robbins et al., 1992; Steele et al., 2023). A subsequent study from the Palmer and Hiiemae group explored pharyngeal phase timing measures prior to and during upper esophageal sphincter opening (UESO) in small samples of healthy adults; longer timing measures for shortbread cookies were found compared to banana and soft tofu stimuli (Hiraoka et al., 2017). These studies predate implementation of the IDDSI framework, and therefore do not enable us to understand how swallowing may vary across different textures, as defined by IDDSI.

As part of our program of research exploring the influence of bolus consistency on healthy swallowing, our group recently reported preliminary data for a nonradiographic study comparing chewing and oral processing across three different RG7 food stimuli: carrots, a round gummy candy, and a cracker (Bandini et al., 2022). The data, collected from a pilot sample of younger adults with no history of dysphagia, suggested that oral processing of these foods resulted in boluses with properties matching IDDSI Level SB6 or lower at the point of transfer to the pharynx (Bandini et al., 2022). Subsequently, the same participants underwent videofluoroscopy with food stimuli representing IDDSI levels MM5, SB6, and RG7. A first analysis of those data (Gandhi et al., 2024) found no significant consistency differences in the following measures of swallow onset patterns or timing: bolus location at swallow onset; swallow reaction time (SRT), that is, the interval from bolus passing mandible (BPM) to hyoid burst (HYB) onset; vallecular aggregation time (VAT), that is, the interval from BPM to end of aggregation (EOA); and the EOA-to-HYB interval. Vallecular aggregation was confirmed to be a common phenomenon, seen in 46% of cases, with a wide interquartile range (IQR) in duration from 66 to 891 ms, extending beyond the HYB frame. By contrast, SRT for these foods was reported to be similar to values previously reported for IDDSI Level 4 stimuli (i.e., extremely thick liquids or pureed foods, EX4/PU4 [Steele et al., 2023]). These findings were interpreted to suggest that the EOA (rather than HYB) might be a preferred event for indexing the beginning of the pharyngeal swallow with chewed foods. The current study involves additional analysis of the same data set (Gandhi et al., 2024), extending that work to characterize swallowing safety, pharyngeal phase timing (subsequent to HYB and EOA), kinematics, and efficiency (i.e., residue) in healthy young adults on IDDSI levels MM5, SB6, and RG7. In order to situate these data relative to prior information for liquid swallowing, we also compared measures for these consistencies to those previously reported reference data for EX4/PU4, obtained using the ASPEKT Method (Analysis of Swallowing Physiology: Events, Kinematics, and Timing), a standard operating procedure for videofluoroscopy rating (Steele et al., 2023). It is important to note that measures relative to EOA are not available for EX4/PU4 given that vallecular aggregation is not an expected phenomenon for liquid stimuli, and also that prior data are drawn from a larger sample of healthy individuals spanning a broad age range (Steele et al., 2023). Given preliminary evidence that the solid food stimuli in this study were likely to have been reduced to a similar consistency after oral processing (Bandini et al., 2022) and the lack of differences in found in swallow onset measures (Gandhi et al., 2024), we adopted the null hypothesis, namely, that pharyngeal phase measures for these foods would be similar to those expected for EX4/PU4 stimuli.

## Method

The data collection protocol for this study has been previously described (Gandhi et al., 2024). Briefly, the study was part of a larger project exploring chewing, food oral processing, and swallowing behaviors in adults without dysphagia across the full range of liquid consistencies and food textures described by the IDDSI Framework (Cichero et al., 2017). Ethics approval for human subjects was obtained from the institutional review board of the University Health Network in Toronto, Canada (UHN CAPCR Protocol 15-9431). The eligibility criteria included:

1. age 18 years or older;
2. intact comprehension to follow two-step commands;
3. no history of previous medical conditions or interventions that could impact swallowing function, including (but not limited to) previous head and neck cancer, prior surgery or radiation to the speech or swallowing apparatus, motor speech diagnoses, respiratory disorders, gastroesophageal disorders, chronic sinusitis, or taste disturbances;
4. no known allergies to any of the stimulus ingredients used in data collection; and
5. not pregnant.

Members of the research team met with each eligible participant to review study procedures prior to obtaining signed informed consent forms and proceeding to the VFSS.

### Videofluoroscopy

VFSS was performed in continuous fluoroscopy mode on an Ultimax ADR-1000 (Toshiba America Medical Systems Inc.) and recorded at 30 frames per second using a calibrated and synchronized TIMS 2000 SP system (TIMS Medical). As previously reported (Gandhi et al., 2024), participants swallowed two boluses of each texture, including barium stimuli prepared in 20% weight-to-volume concentration according to standard recipes (see Supplemental Material S1) to represent IDDSI Levels MM5, SB6, and RG7. Participants were asked to take a teaspoon full of the MM5 stimulus, a single cube of the SB6 stimulus from a container, and a comfortable bite of the RG7 stimulus. The specific instructions were to “take a [comfortable spoonful/cube/comfortable bite] and swallow whenever you are ready.” Mean (± *SD*) values for bolus weight were calculated by bolus consistency using cup weights taken before and after consumption of each MM5 or SB6 bolus. Similarly, bolus weights for the RG7 stimulus were collected during a previous in-lab session where the same cracker with barium paste stimulus was consumed by the same participants.

### Videofluoroscopy Rating

Separate videofluoroscopy clips were recorded for each bolus. These clips were deidentified and randomly assigned to trained raters for rating according to the ASPEKT Method (Steele et al., 2023). This method involves duplicate blinded rating according to clear operational definitions for measurement components (see Supplemental Material S2). For this study, each clip was rated by two raters from a team of five trained and calibrated raters. Discrepancies exceeding a priori established criteria were flagged and sent to a meeting for resolution by consensus. Prior to discrepancy resolution, intraclass correlations were calculated for reliability for all measurements.

To derive timing measures, key frames were identified on the initial swallow of each bolus. For the current analysis, these included frames corresponding to the end of bolus aggregation in the valleculae (EOA), onset of the HYB, LVC, UESO, maximum pharyngeal constriction (MPC), maximum UES distention (UESMaxFrame), UES closing (UESC), laryngeal vestibule closure offset, and a swallow rest (SR) frame at the end of the initial swallow for the bolus. Additional details regarding the definitions of these events can be found in Supplemental Material S2. With these key frames confirmed, the following timing measures were derived in frames:

- latencies to UESO and LVC relative to two onset events: HYB and EOA; and
- LVC and UESO duration (i.e., the difference between onset and offset frames for these events).

Swallowing safety was scored on every bolus using the 8-point Penetration–Aspiration Scale (Rosenbek et al., 1996). With respect to measures of kinematics and efficiency, pixel-based tracing was used to measure the following, with all measures normalized to a (C2-4) cervical spine scalar (Molfenter & Steele, 2014):

- pharyngeal area on the MPC (PhAMPC) frame;
- UES maximum distention (UESMAX) on the UESMaxFrame; and
- postswallow residue area in the valleculae, pyriform sinuses, elsewhere in the pharynx, and the sum of these areas (total pharyngeal residue) on the SR frame.

### Statistical Analysis

All statistical analyses were performed in SPSS (Version 29), with statistical significance defined as *p* < .05. Preconsensus reliability for event identification was assessed by calculating the absolute frame difference between ratings and determining the frequency of discrepancies exceeding an a priori criterion of three frames for all events prior to UESC and to five frames for UESC and SR. These thresholds were established according to the routine procedure in our lab, which involves inspection of the distribution of rater disagreements across rating batches and identifies at least the top 10% of that distribution for review (Steele et al., 2022). Two-way mixed-model intraclass correlation coefficients (ICCs) for average measures of consistency were also calculated for event identification and pixel-based measures. Mean parameter values were calculated for each participant across the two task repetitions for each texture, with timing values rounded to the closest whole integer frame and pixel-based measures rounded to the closest single decimal place value. Descriptive statistics (median and IQR boundaries) were then calculated for each parameter based on participant mean values. Given nonnormal distributions of residuals for multiple parameters, nonparametric Friedman's tests were used to identify significant differences by texture (i.e., MM5, SB6, RG7) with post hoc testing of pairwise comparisons using Wilcoxon signed-ranks tests. Box plots and one-sample *t* tests were then used to compare means and standard deviations to previously reported medians for teaspoon-sized boluses of EX4/PU4 (Steele et al., 2023).

## Results

The study sample included 20 adults without dysphagia (10 men, 10 women), aged 23–55 years (*M* = 27.8, *SD* = 7.5). With regard to race, two participants identified as Black/African American, four as Asian, and the remaining participants as White. None of the participants identified as Hispanic.

### Reliability

Preconsensus event identification fell within the established tolerance for nondiscrepant ratings across the two blinded ratings 76.2% of the time. ICCs for event identification showed good preconsensus reliability, with an ICC of .89, 95% confidence interval [CI] [.88, .91]. Similarly, ICCs for pixel-based measures showed excellent preconsensus reliability (ICC = .93, 95% CI [.91, .94]).

### Bolus Weights

*M* ± *SD*s for bolus weight by consistency were as follows: MM5: 8.84 ± 3.2 g; SB6: 6.25 ± 1.3 g; RG7: 4.64 ± 2.7 g. The Friedman's tests identified significant differences across these consistencies for bolus weight: χ^2^(2) = 19.9, *p* < .001. Post hoc Wilcoxon tests identified significantly larger bolus weights for MM5 compared to both SB6 and RG7 (*z* ≥ 2.84, *p* < .01), and significantly larger bolus weights for SB6 than for RG7 (*z* = 3.13, *p* < .01). These weights compare to previously reported mean bolus weights for teaspoon-administered boluses of EX4/PU4 stimuli of 5.97 ± 3.0 g (5.15 ± 2.6 ml; Steele et al., 2019). *t* tests showed that the MM5 boluses were significantly heavier, *t*(19) = 4.43, *p* < .001, Cohen's *d* = 0.99, large effect, and the RG7 boluses were significantly lighter, *t*(19) = −3.67, *p* < .001, Cohen's *d* = −0.82, large effect, than the reported values for EX4/PU4 boluses, while no differences in weight were found for the SB6 stimuli.

### Timing

**Figure 1.**
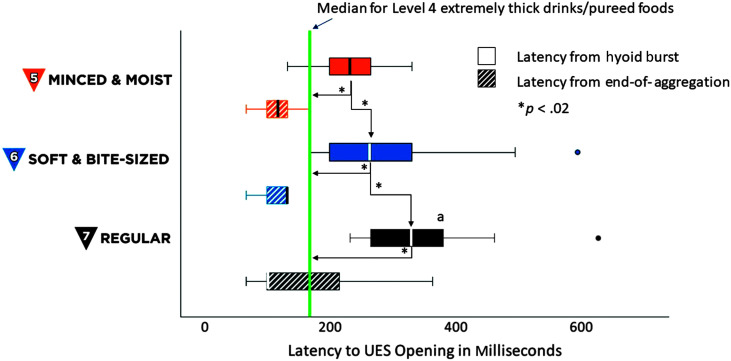
Latencies from hyoid burst (HYB) onset and end of aggregation (EOA) until upper esophageal sphincter (UES) opening. The box plot shows latencies from HYB onset and EOA until UES opening, in milliseconds, for International Dysphagia Diet Standardisation Initiative (IDDSI) Food Levels MM5 (minced and moist), SB6 (soft and bite-sized) and RG7 (regular). Standard IDDSI colors are used for each level: blue for MM5, orange for SB6, and black for RG7. Median values are shown by the bold lines within each box, with the boundaries of each box marking the 25th and 75th percentiles. Outlier values are shown by small dots falling beyond the boundaries of the whiskers. For comparison, the previously reported median value for IDDSI Level 4 (extremely thick liquids/pureed foods) is shown by the green reference line. Significant differences were seen for all comparisons indexed relative to HYB. There were no significant differences when latencies were indexed relative to EOA.

**Figure 2.**
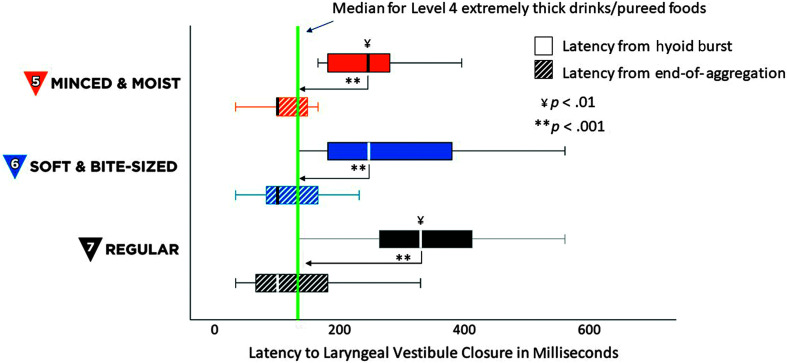
Latencies from hyoid burst (HYB) onset and end of aggregation (EOA) until laryngeal vestibule closure. The box plot shows latencies from HYB onset and EOA until laryngeal vestibule closure, in milliseconds, for International Dysphagia Diet Standardisation Initiative (IDDSI) Food Levels MM5 (minced and moist), SB6 (soft and bite-sized), and RG7 (regular). Standard IDDSI colors are used for each level: blue for MM5, orange for SB6, and black for RG7. Median values are shown by the bold lines within each box, with the boundaries of each box marking the 25th and 75th percentiles. Outlier values are shown by small dots falling beyond the boundaries of the whiskers. For comparison, the previously reported median value for IDDSI Level 4 (extremely thick liquids/pureed foods) is shown by the green reference line. Significant differences were seen for several comparisons indexed relative to HYB. There were no significant differences when latencies were indexed relative to EOA.

**Figure 3.**
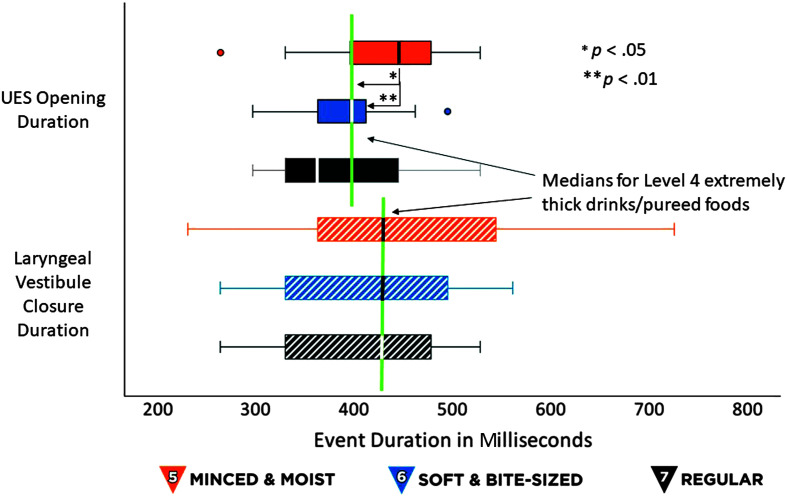
Durations of upper esophageal sphincter (UES) opening and laryngeal vestibule closure (LVC). The box plot shows durations for UES opening (UESO) and LVC, in milliseconds, for International Dysphagia Diet Standardisation Initiative (IDDSI) Food Levels MM5 (minced and moist), SB6 (soft and bite-sized), and RG7 (regular). Standard IDDSI colors are used for each level: blue for MM5, orange for SB6, and black for RG7. Median values are shown by the bold lines within each box, with the boundaries of each box marking the 25th and 75th percentiles. Outlier values are shown by small dots falling beyond the boundaries of the whiskers. For comparison, the previously reported median value for IDDSI Level 4 (extremely thick liquids/pureed foods) is shown by the green reference line. Significant texture effects were seen for UESO duration between MM5 stimuli and both SB6 stimuli and previously reported values for EX4/PU4 stimuli. There were no significant effects of texture on LVC duration.

Descriptive statistics for the swallow timing measures are shown in Table 1. Latencies from HYB to UESO increased significantly across textures: MM5 < SB6 < RG7, χ^2^(2) = 19.75, *p* < .001. All pairwise comparisons were significant (*z* ≥ 2.29, *p* ≤ .02) and values for all three consistencies were significantly longer than those previously reported for EX4/PU4, *t*(19) ≥ 4.53, *p* < .001, Cohen's *d* = 1.01, (large effect). However, there were no significant effects of texture when latencies to UESO were calculated relative to EOA, noting that EOA has not previously been reported for EX4/PU4 stimuli. These results are illustrated in Figure 1. Latencies from HYB to LVC increased significantly across textures: MM5 < SB6 < RG7, χ^2^(2) = 14.71, *p* < .001, with a single significant pairwise comparison between MM5 and RG7 (*z* = 3.26, *p* < .01). Values for all three consistencies were significantly longer than those previously reported for EX4/PU4, *t*(19) ≥ 5.36, *p* < .001, Cohen's *d* = 1.2, (large effect). However, there were no significant effects of texture when latencies to LVC were calculated relative to EOA, as illustrated in Figure 2. UESO duration was significantly longer for MM5 stimuli than for SB6 and RG7, χ^2^(2) = 9.63, *p* < .01, with a single significant pairwise comparison between MM5 and SB6, *z* = 3.14, *p* < .01. Values for MM5 were also significantly longer than those reported for EX4/PU4 stimuli, *t*(19) = 1.94, *p* < .05, Cohen's *d* = 0.43, (medium effect), as illustrated in the top half of Figure 3. There were no significant effects of texture on LVC duration, either across MM5, SB6 and RG7, or in comparison to previously reported values for EX4/PU4, as shown in the bottom half of Figure 3.

**Table 1.** Descriptive statistics for timing measures by consistency.

Parameter	Consistency	In frames (at 30 frames/second)			In milliseconds (at 33 ms/frame)			Significant pairwise comparisons (*p* < .05)
		*Mdn*	p25	p75	*Mdn*	p25	p75	
Hyoid burst to UES opening	Minced & moist (MM5)	7	6	8	231	198	264	EX4/PU4 < MM5 < SB6 < RG7
	Soft & bite-sized (SB6)	8	6	10	264	198	330	
	Regular (RG7)	10	8	12	330	264	388	
	Extremely thick liquids/pureed foods (EX4/PU4)^a^	5	4	6	165	132	198	
End of aggregation to UES opening	Minced & moist (MM5)	4	3	4	116	99	132	None
	Soft & bite-sized (SB6)	4	3	4	132	99	132	
	Regular (RG7)	3	3	7	99	99	223	
	Extremely thick liquids/pureed foods (EX4/PU4)^a^	Not reported						
Time to most complete LVC	Minced & moist (MM5)	8	5	9	248	173	289	EX4/PU4 < thicker textures; MM5 < RG7
	Soft & bite-sized (SB6)	8	5	12	248	173	388	
	Regular (RG7)	10	8	13	330	264	421	
	Extremely thick liquids/pureed foods (EX4/PU4)^a^	4	3	5	132	99	165	
End of aggregation to LVC	Minced & moist (MM5)	3	3	5	99	99	157	None
	Soft & bite-sized (SB6)	3	2	6	99	74	182	
	Regular (RG7)	3	2	6	99	66	190	
	Extremely thick liquids/pureed foods (EX4/PU4)^a^	Not reported						
UES opening duration	Minced & moist (MM5)	14	12	15	446	396	487	MM5 > EX4/PU4; MM5 > SB6
	Soft & bite-sized (SB6)	12	11	13	396	363	421	
	Regular (RG7)	11	10	14	363	330	454	
	Extremely thick liquids/pureed foods (EX4/PU4)^a^	12	11	13	396	363	429	
LVC duration	Minced & moist (MM5)	13	11	17	429	363	553	None
	Soft & bite-sized (SB6)	13	10	15	429	314	495	
	Regular (RG7)	13	10	15	429	330	487	
	Extremely thick liquids/pureed foods (EX4/PU4)^a^	13	12	14	429	396	462	

*Note.* UES = upper esophageal sphincter; LVC = laryngeal vestibule closure; p25 = 25th percentile; p75 = 75th percentile.

a Previously reported in Steele et al. (2023).

### Swallowing Safety, Kinematics, and Efficiency

**Figure 4.**
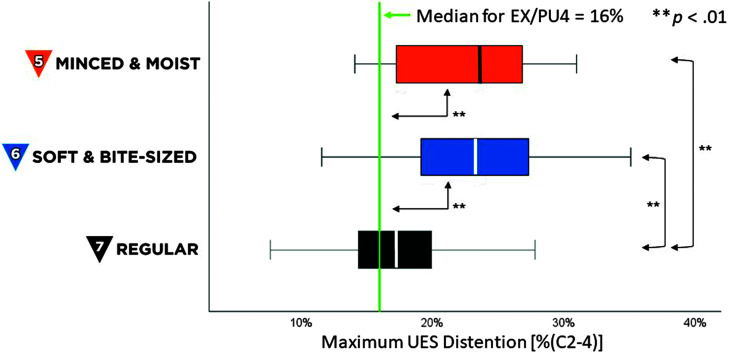
Maximum upper esophageal sphincter (UES) distention. The box plot shows measures of maximum UES distention, in %(C2-4) units, for International Dysphagia Diet Standardisation Initiative (IDDSI) Food Levels MM5 (minced and moist), SB6 (soft and bite-sized), and RG7 (regular). Standard IDDSI colors are used for each level: blue for MM5, orange for SB6, and black for RG7. Median values are shown by the bold lines within each box, with the boundaries of each box marking the 25th and 75th percentiles. Outlier values are shown by small dots falling beyond the boundaries of the whiskers. For comparison, the previously reported median value for IDDSI Level 4 (extremely thick liquids/pureed foods) is shown by the green reference line.

**Figure 5.**
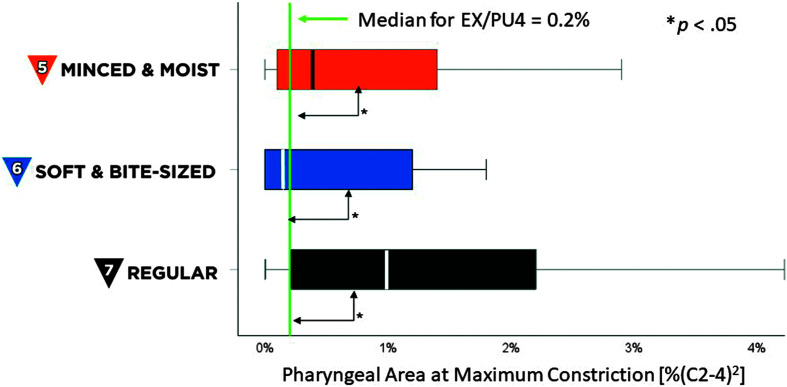
Pharyngeal area at maximum constriction. The box plot shows measures of pharyngeal area at maximum constriction, in %(C2-4)2 units, for International Dysphagia Diet Standardisation Initiative (IDDSI) Food Levels MM5 (minced and moist), SB6 (soft and bite-sized), and RG7 (regular). Standard IDDSI colors are used for each level: blue for MM5, orange for SB6, and black for RG7. Median values are shown by the bold lines within each box, with the boundaries of each box marking the 25th and 75th percentiles. Outlier values are shown by small dots falling beyond the boundaries of the whiskers. For comparison, the previously reported median value for IDDSI Level 4 (extremely thick liquids/pureed foods) is shown by the green reference line.

Maximum penetration–aspiration scale scores for all boluses in this project were 1 (i.e., no airway invasion). Descriptive statistics for measures of swallowing kinematics and efficiency are shown in Table 2. UESMaxFrame values for RG7 foods were similar to those previously reported for EX4/PU4 stimuli, as shown by the green line in Figure 4. MM5 and SB6 foods showed significantly wider UES distention than the RG7 foods tested in this study, χ^2^(2) = 12.9, *p* < .01, z ≥ 2.76, *p* < .01, and compared to previously reported values for EX4/PU4 stimuli, *t*(19) ≥ 5.7, *p* < .001, Cohen's *d* = 1.2, (large effect). Although there were no significant differences in pharyngeal area at maximum constriction across MM5, SB6, and RG7 foods, the values for MM5, SB6, and RG7 stimuli showed significantly larger pharyngeal area compared to previously reported reference values for EX4/PU4 stimuli, as represented by the green line in Figure 5, *t*(19) ≥ 1.9, *p* < .05, Cohen's *d* ≥ 0.43, (small effect).

**Table 2.** Descriptive statistics for pixel-based measures by consistency.

Parameter [unit]	Consistency	*Mdn*	p25	p75
Maximum UES distention [%(C2-4)]	Minced & moist (MM5)	23.6	17.2	26.9
	Soft & bite-sized (SB6)	23.3	19.1	27.4
	Regular (RG7)	17.4	14.4	20.0
	Extremely thick liquids/pureed foods (EX4/PU4)^a^	16.0	14.0	18.0
Pharyngeal area at maximum pharyngeal constriction [%(C2-4)^2^]	Minced & moist (MM5)	0.0	0.5	1.3
	Soft & bite-sized (SB6)	0.0	0.0	0.9
	Regular (RG7)	0.0	0.8	1.7
	Extremely thick liquids/pureed foods (EX4/PU4)^a^	0.2	0.0	1.4
Vallecular residue [%(C2-4)^2^]	Minced & moist (MM5)	0.0	0.1	0.6
	Soft & bite-sized (SB6)	0.0	0.0	0.1
	Regular (RG7)	0.0	0.2	0.6
	Extremely thick liquids/pureed foods (EX4/PU4)^a^	0.0	0.0	0.5
Pyriform sinus residue [%(C2-4)^2^]	Minced & moist (MM5)	0.0	0.1	0.3
	Soft & bite-sized (SB6)	0.0	0.0	0.0
	Regular (RG7)	0.0	0.0	0.3
	Extremely thick liquids/pureed foods (EX4/PU4)^a^	0.0	0.0	0.5
Other pharyngeal residue [%(C2-4)^2^]	Minced & moist (MM5)	0.0	0.1	0.3
	Soft & bite-sized (SB6)	0.0	0.0	0.1
	Regular (RG7)	0.0	0.1	0.4
	Extremely thick liquids/pureed foods (EX4/PU4)^a^	0.1	0.0	0.6
Total pharyngeal residue [%(C2-4)^2^]	Minced & moist (MM5)	0.0	0.4	1.5
	Soft & bite-sized (SB6)	0.0	0.0	0.3
	Regular (RG7)	0.1	0.7	1.5
	Extremely thick liquids/pureed foods (EX4/PU4)^a^	0.1	0.0	1.5

*Note.* p25 = 25th percentile; p75 = 75th percentile.

a Previously reported in Steele et al. (2023).

**Figure 6.**
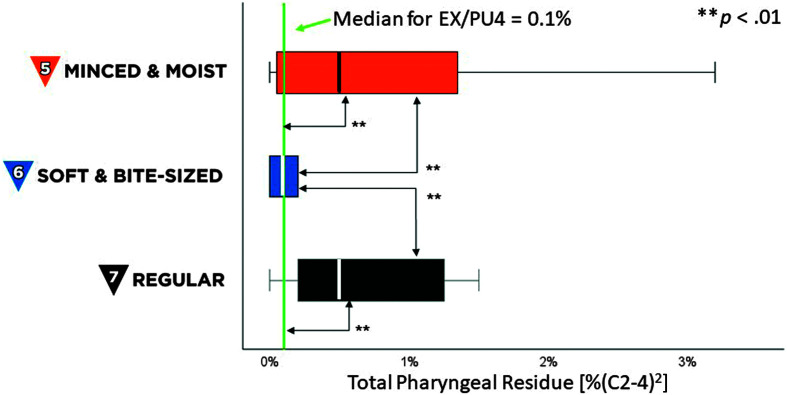
Total pharyngeal residue. The box plot shows measures of total pharyngeal residue, in %(C2-4)2 units, for International Dysphagia Diet Standardisation Initiative (IDDSI) Food Levels MM5 (minced and moist), SB6 (soft and bite-sized), and RG7 (regular). Standard IDDSI colors are used for each level: blue for MM5, orange for SB6, and black for RG7. Median values are shown by the bold lines within each box, with the boundaries of each box marking the 25th and 75th percentiles. Outlier values are shown by small dots falling beyond the boundaries of the whiskers. For comparison, the previously reported median value for IDDSI Level 4 (extremely thick liquids/pureed foods) is shown by the green reference line.

**Figure 7.**
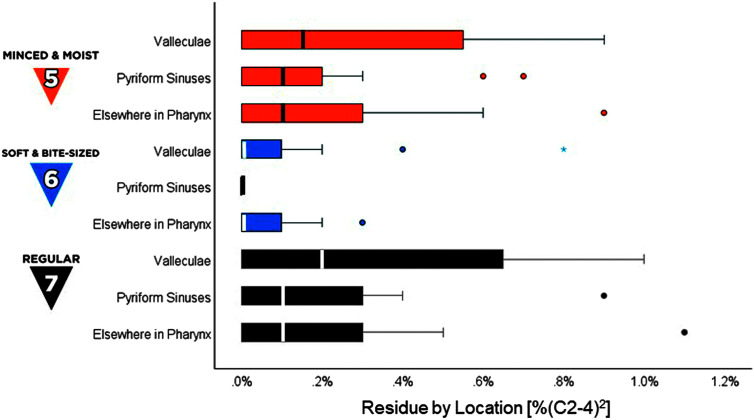
Pharyngeal residue by location. The box-plot shows measures of pharyngeal residue, by location, in %(C2-4)2 units, for International Dysphagia Diet Standardisation Initiative (IDDSI) Food Levels MM5 (minced and moist), SB6 (soft and bite-sized), and RG7 (regular). Standard IDDSI colors are used for each level: blue for MM5, orange for SB6, and black for RG7. Median values are shown by the bold lines within each box, with the boundaries of each box marking the 25th and 75th percentiles. Outlier values are shown by small dots falling beyond the boundaries of the whiskers.

For total pharyngeal residue, values for SB6 foods were significantly smaller than those seen for MM5 and RG7 foods, χ^2^(2) = 20.96, *p* < .001, *z* ≥ 3.33, *p* < .01. Residue for MM5 and RG7 foods were also significantly larger than previously reported values for teaspoons of EX4/PU4 stimuli, *t*(19) ≥ 3.1, *p* < .01, Cohen's *d* ≥ 0.7, (medium effect), as shown in Figure 6. For residue by location, median values for all locations were 0% (C2-4)^2^ across MM5, SB6, and RG7 foods, compared to previously reported medians between 0% and 0.1% (C2-4)^2^ for EX4/PU4 stimuli. No pyriform sinus residue was observed with soft and bite-sized boluses. Residue for MM5 and RG7 foods was predominantly located in the valleculae (see Figure 7).

## Discussion

In this study, we explored differences in quantitative videofluoroscopic measures of swallow safety, timing, kinematics, and efficiency for boluses representing IDDSI food levels MM5, SB6, and RG7, comparing values across these levels and relative to previously reported data for IDDSI level EX4/PU4. In the absence of previously reported data for these consistencies, we adopted the null hypothesis for all tests. The analysis did not support this hypothesis; rather, significant variations were seen across these stimuli for several parameters, suggest that differences in bolus properties influence the pharyngeal phase of swallowing. These differences may include differences in particle size, bolus cohesiveness and bolus volume.

A key finding of this study is recognition that although pharyngeal stage duration from HYB to UESO shows significant stepwise increases from IDDSI level EX4/PU4 to IDDSI level RG7, when timing measures are indexed relative to EOA rather than HYB, latencies to UESO do not differ by consistency. There are two implications of this finding. First, as suggested in the previous paper (Gandhi et al., 2024), EOA may be a more appropriate event than HYB to use as a marker of pharyngeal swallow initiation with foods. Second, the data suggest that the Stage II transport phenomenon of bolus aggregation in the valleculae varies across food textures and that the time window between BPM and EOA, during which aliquots of processed food are transported into the pharynx, is likely where texture-based variations occur. The current data point to this conclusion, even though the previous analysis failed to identify significant differences across textures in SRT, VAT, or EOA–HYB (Gandhi et al., 2024). Closer inspection of those previous data shows that HYB occurred prior to EOA across all consistencies tested, with an overall median latency of −132 ms (i.e., four frames) and IQR of −231 to −83 ms (Gandhi et al., 2024). Notably, very large pooled IQRs were reported for SRT (−66 to 743 ms) and VAT (66 to 891 ms), with 75th percentile values for individual consistencies ranging from 743 to 1,060 ms for SRT and from 825 to 1,044 ms for VAT. Additionally, neither the prior nor the current analysis documented the number of separate bolus transport events during aggregation, or the timing of these events relative to cyclic jaw movements, both of which may be metrics of interest for future studies. The current experiment is not adequately powered, either in terms of sample size or with respect to the number of stimuli and task repetitions included in the protocol to provide greater insights regarding this variation. Additional research is needed to confirm expected latencies between solid bolus arrival in the pharynx and swallow initiation, and to distinguish normal aggregation from situations suggesting clinical concern in the form of premature bolus spillage or delayed pharyngeal swallow initiation. If comparison of data across VFSS and fiberoptic endoscopic evaluation of swallowing (FEES) can establish similar timing values and consistency-based trends, this would open the door to more detailed exploration across a wider variety of food stimuli using FEES without concerns related to radiation exposure.

The other timing measure showing significant differences across bolus consistency was the duration of UES opening. Here, the MM5 boluses elicited significantly longer durations of opening than the SB6 stimuli and previously reported values for EX4/PU4 stimuli. Interestingly, the MM5 boluses, together with the SB6 boluses also showed greater distention of the UES compared to RG7 and EX4/PU4. These findings raise questions about the degree to which variations in bolus size may have contributed to the observed variations in UES distention, given that previous research on liquid swallows strongly suggests that UES distention and residue vary with bolus size (Cook et al., 1989; Jacob et al., 1989; Kahrilas et al., 1988, 1995, 1996; Kern et al., 1999). In this study, we allowed participants to self-select bolus size for MM5 (teaspoon) and RG7 (bite), while a single 1.5 cm^3^ cube of the SB6 stimulus was taken. These methods mirror the comfortable teaspoon protocol used in our prior reference values work for EX4/PU4 stimuli (Steele et al., 2019, 2023) but differ from the common approach across the food science literature of matching samples by weight (e.g., Cichero et al., 2013; Devezeaux de Lavergne et al., 2017; Fontijn-Tekamp et al., 2004; Hoebler et al., 2000; Mishellany et al., 2006; Peyron et al., 2011). Cup weights were used to determine bolus size in this experiment, and showed that the MM5 boluses weighed significantly more than the SB6 boluses, with these being similar in weight to teaspoons of EX4/PU4, and heavier than the RG7 stimuli. The fact that the MM5 boluses were heavier than other stimuli in this study does appear to correspond to the longer UES opening durations and wider UES distention seen with the MM5 stimulus. Patterns of bolus weight do not, however, align fully with the patterns seen in UES measures across the full range of stimuli tested.

To further explore the issue of bolus size (i.e., weight) influences on UES measures, we performed post hoc analyses, for which the measures of UES opening duration and distention were divided by group mean values for bolus weight for the consistencies tested. For UESO duration per gram, significant stepwise increases were seen from MM5 < SB6 < RG7, χ^2^(2) = 38.1, *p* < .001; *z* ≥ 3.85, *p* < .01. Similarly, for UES distention per gram, significant stimulus differences were seen, χ^2^(2) = 24.7, *p* < .001, with wider opening for RG7 compared to both MM5 and SB6 (*z* ≥ 2.7, *p* < .01). These results highlight the fact that measures of bolus or sample weight reflect the amount taken into the mouth, prior to oral processing, but may not necessarily accurately reflect either the weight or the size of the bolus that eventually passes through the UES. We speculate that differences in other bolus properties, including size, shape, and cohesiveness at the point of transit through the UES may have contributed to the observed differences in UES parameters. Additional research is needed to elucidate this question further.

Differences in bolus size and cohesiveness may also contribute to differences in PhAMPC and pharyngeal residue. In the current study, these differences had smaller effect sizes than the observed differences in UES parameters. Nevertheless, the data for SB6 boluses stand out as interesting, with similar pharyngeal constriction but better efficiency and less residue compared to MM5 and RG7 foods. Here, post hoc explorations of weight-adjusted values for PhAMPC revealed a pattern of significantly larger PhAMPC per gram for RG7 compared to MM5 and SB6, χ^2^(2) = 9.8, *p* < .01; *z* ≥ 2.6, *p* < .01. Measures of total residue per gram also showed significant differences across consistency, χ^2^(2) = 19.8, *p* < .01, with the smaller amounts of residue for SB6 than for MM5 (*z* = 3.01, *p* < .01) and a further significant increase for the RG7 stimuli (*z* = 2.4, *p* < .05).

These findings raise an important clinical question with regard to the stimuli that are included in protocols used in videofluoroscopy, namely, should food stimuli be tested routinely, and if yes, of what texture(s)? Currently, there are no standard, regulatorily approved barium stimuli for the food side of the IDDSI Framework. Furthermore, it has been argued that videofluoroscopy is not an ideal test to use for exploring differences in swallowing across food textures in clinical practice given the importance of limiting radiation dose to the patient (Steele et al., 2021). Inclusion of at least one solid bolus (typically a cracker or cookie) is common in widely used videofluoroscopy protocols including the Modified Barium Swallow Impairment Profile (Martin-Harris et al., 2008) and the protocol used in development of the Dynamic Imaging Grade of Swallowing Toxicity (DIGEST; Hutcheson et al., 2017, 2022). Furthermore, residue with cracker stimuli appears to commonly be score driving with respect to the DIGEST efficiency grade (Hutcheson et al., 2022). In healthy adults, the current experiment showed that the RG7 stimulus (i.e., a bite of cracker smeared with extremely thick barium) elicited similar post-aggregation timing measures to reported values for teaspoons of EX4/PU4, as well as similar UES distention and timing. However, smaller (i.e., better) reported values for both pharyngeal constriction and residue are published for EX4/PU4 than those seen in this experiment with the RG7 cracker. The fact that the cracker boluses were of smaller weight than teaspoons of EX4/PU4 may amplify these differences. By comparison, teaspoons of MM5 stimuli appear to elicit wider and longer durations of UES opening but poorer pharyngeal constriction and more residue than teaspoons of Level 4 EX4/PU4 foods. Thus, these data suggest that teaspoons of EX4/PU4 stimuli do not provide sufficient data to predict swallowing physiology or efficiency for higher levels on the IDDSI framework. It is interesting to note that the SB6 stimuli appear to show both optimal UES opening and the least residue across the range of foods that were tested in this study. Taken together, these results appear to support routine inclusion of an RG7 task in videofluoroscopy protocols to challenge pharyngeal constriction and efficiency and inclusion of SB6 as a compensatory intervention to optimize swallowing efficiency. It must, however, be noted that a cracker smeared with barium paste is only one possible example of an RG7 stimulus, and that the RG7 level on the IDDSI framework contains an essentially infinite variety of regular foods. Whether routine testing of different RG7 food stimuli, or of nonfood stimuli such as barium-filled gelatin pill capsules might reveal different types of impairment remains unknown. Further work on establishing optimal food stimuli for videofluoroscopy is definitely needed.

### Limitations

The preceding discussion highlights several acknowledged limitations of this study, specifically with regard to the small selection of stimuli representing the different IDDSI food levels, and the issue of differences in bolus size, shape, and cohesiveness being factors that were not controlled. Additionally, it is important to recognize that the sample for this study was small (*n* = 20) and composed of healthy younger adults (≤ 55 years of age). As such, the study was not sufficiently powered to allow comparisons across sex nor to consider age as a parameter of interest. Furthermore, only two boluses of each consistency were tested; for some parameters (particularly those prior to EOA), considerable variation was seen across these two boluses, suggesting that further study is needed to understand within-participant variations in swallowing across repeated presentations of food stimuli. Finally, although the experimental stimuli were prepared to match IDDSI definitions, future research would be needed to confirm that other stimuli within the same IDDSI levels elicit similar behaviors.

## Conclusions

This study extends our understanding of oropharyngeal swallowing physiology across different food textures in healthy younger adults. Future research is needed to explore these measures across a wider age range. Three key messages emerge for current clinical practice. First, EOA appears to be a more suitable event to index the beginning of the pharyngeal phase of swallowing when calculating timing measures with food stimuli. Second, the study highlights several parameters where texture influences may interact with bolus size or weight. Further research is required to elucidate this issue. Third, the data suggest that data for pureed food or extremely thick liquid stimuli do not provide sufficient information to predict swallowing measures with food stimuli. Inclusion of a RG7 stimulus such as a cracker is recommended for VFSS protocols, noting that this task appears to provide additional information regarding pharyngeal constriction and swallowing efficiency. We emphasize that the recipes developed for this study are off-label mixtures of barium sulfate powder with food ingredients, and urge caution in generalizing from the current results to different examples of stimuli in IDDSI Levels 5, 6, and especially Level 7 (which contains an infinite variety of nonmodified food textures). Additionally, we urge caution in generalizing from the current results to different bolus sizes or volumes. Future work will be needed to try to determine how differences in the duration, force, and efficiency of chewing and oral processing influence both bolus characteristics and pharyngeal swallowing metrics.

## Author Contributions

**Pooja Gandhi:** Formal analysis (Supporting), Writing – original draft (Equal), Writing – review & editing (Supporting). **Emily Barrett:** Investigation (Supporting), Data curation (Supporting), Writing – review & editing (Supporting). **Renata Mancopes:** Investigation (Supporting), Data curation (Supporting), Writing – review & editing (Supporting). **Vanessa Panes:** Investigation (Supporting), Data curation (Supporting), Writing – review & editing (Supporting). **Melanie Peladeau-Pigeon:** Data curation (Lead), Investigation (Supporting), Project administration (Supporting), Software (Lead), Validation (Lead), Writing – review & editing (Supporting). **Michelle M. Simmons:** Investigation (Supporting), Data curation (Supporting), Writing – review & editing (Supporting). **Catriona M. Steele:** Conceptualization (Lead), Formal analysis (Lead), Funding acquisition (Lead), Investigation (Lead), Methodology (Lead), Project administration (Lead), Resources (Lead), Supervision (Lead), Visualization (Lead), Writing – original draft (Equal), Writing – review & editing (Lead).

## Data Availability Statement

The data sets generated during and/or analyzed during the current study are not publicly available due to ethical/legal restrictions. Inquiries regarding access to the data should be directed to the corresponding author.

## Supplementary Material

10.1044/2025_JSLHR-25-00546SMS1 Supplemental Material S1 Recipes used for preparing food stimuli with barium contrast.

10.1044/2025_JSLHR-25-00546SMS2 Supplemental Material S2 Abbreviations and definitions used in VFSS rating according to the ASPEKT Method (Analysis of Swallowing Physiology: Events, Kinematics and Timing) (Steele et al., 2023).
