# Supplementary material for: What About Foods? The Influence of Food Texture on the Safety, Timing, Kinematics, and Efficiency of Pharyngeal Phase Swallowing in Healthy Adults
Source: J Speech Lang Hear Res. 2026 Mar 26;69(4):1528–41. doi: 10.1044/2025_JSLHR-25-00546 (PMC13081152; doi:10.1044/2025_JSLHR-25-00546)
Supplement: Supplemental Material S1 [file JSLHR-69-1528-s001.pdf]

Supplemental Material S1. Recipes used for preparing food stimuli with barium contrast.

Radio-opaque food stimuli for IDDSI Food Levels 5 (Minced-and-moist), 6 (Soft and Bite-Sized) and 7 (Regular) were prepared according to the following recipes:

IDDSI Level 5 Minced-and-moist Food:

1. Create 125 ml of a 30% w/v liquid barium solution using 39 grams of Bracco E-ZPaque® Powder and 116 grams of bottled water. Mix using a kitchen stand mixer for 2 minutes 30 seconds.
2. In a separate container, combine 15.92 of breadcrumbs (NuCibo Texture Modified Bread & Bakery Mix) and ½ tablespoon (7.5 ml) of cocoa powder (Fry’s* Premium Cocoa).
3. Measure 3.7 ml of canola oil (Longo’s brand) using a syringe and add to the breadcrumb and cocoa powder mixture.
4. Add 60 ml of the 30% w/v liquid barium solution to the breadcrumb, cocoa powder and oil mixture. Stir manually until evenly blended.

IDDSI Level 6 Soft and Bite-sized Food:

1. Create 125 ml of a 40% w/v liquid barium solution using 52 grams of Bracco E-ZPaque® Powder and 113 grams of bottled water. Mix using a kitchen stand mixer for 2 minutes 30 seconds.
2. In a separate container, combine 15.92 of breadcrumbs (NuCibo Texture Modified Bread & Bakery Mix) and ½ tablespoon (7.5 ml) of cocoa powder (Fry’s* Premium Cocoa).
3. Measure 3.7 ml of canola oil (Longo’s brand) using a syringe and add to the breadcrumb and cocoa powder mixture.
4. Add 30 ml of the 30% w/v liquid barium solution to the breadcrumb, cocoa powder, and oil mixture. Stir manually until evenly blended.
5. Allow the mixture to sit for 10 minutes to allow it to solidify.
6. Using gloves, remove ½ teaspoon portions from the mixture and shape into cubes of 1.5 cm3. Serve by teaspoon.

IDDSI Level 7 Regular Food:

This consistency is prepared by smearing a barium paste onto a Carr’s® Table Water® Cracker.

To create the barium paste, follow these instructions:

1. Create one cup of paste using 108 grams of Bracco E-Z-Paque® Powder and a full 108 g container of Kraft Jell-O chocolate pudding. Stir manually until well blended.
2. Smear the chocolate barium paste onto the cracker just prior to use.
