# Supplementary material for: What About Foods? The Influence of Food Texture on the Safety, Timing, Kinematics, and Efficiency of Pharyngeal Phase Swallowing in Healthy Adults
Source: J Speech Lang Hear Res. 2026 Mar 26;69(4):1528–41. doi: 10.1044/2025_JSLHR-25-00546 (PMC13081152; doi:10.1044/2025_JSLHR-25-00546)
Supplement: Supplemental Material S2 [file JSLHR-69-1528-s002.pdf]

Supplemental Material S2. Abbreviations and definitions used in VFSS rating according to the ASPEKT Method (Analysis of Swallowing Physiology: Events, Kinematics and Timing) (Steele et al., 2023).

a) Events:

BPM_frame	Bolus passing the ramus of the mandible: The first frame where the leading edge of the bolus touches or crosses the shadow of the inferior ramus of the mandible.
EOA_frame	End of bolus aggregation in the valleculae, marked by the first frame showing movement of the leading edge of the bolus out of the vallecular space
HYB_frame	Hyoid burst onset: The first frame associated with the initial bolus swallow showing an anterior-superior jump in hyoid position (i.e., with a larger magnitude of displacement across the two frames than in preceding position changes).
LVC_frame	Laryngeal vestibule closure: The first frame of most-complete laryngeal vestibule closure associated with the initial bolus swallow (i.e., showing the least amount of contrast/air in the laryngeal vestibule, anterior to the arytenoids).
UESO_frame	Upper esophageal sphincter opening: The first frame where the leading edge of the bolus (or in rare cases, air) enters into the top of the upper esophageal sphincter (UES).
UESMax_frame	Frame of maximum UES distention: The frame where the opening of the UES shows the greatest distention (i.e., widest lumen width and/or bolus column).
MPC_frame	Maximum Pharyngeal Constriction: The first frame associated with the initial bolus swallow showing the least amount of bolus flow and/or airspace in the pharynx.

LVCOff_frame Laryngeal Vestibule Closure Offset: The first frame where there is visible opening of the laryngeal vestibule after the LVC_frame and relative to its maximum closure, i.e., the arytenoid process moves away from the undersurface of the epiglottis and the space between these structures gets larger.

UESC_frame Upper Esophageal Sphincter Closure: The first frame where the UES achieves closure behind the bolus tail.

SR_frame Swallow Rest: The first frame showing the pyriform sinuses at their lowest position, relative to the spine, within 30 frames (approx. 1 second) of UESC_frame.

b) Timing Measures

SRT Swallow reaction time: HYB_frame-BPM_frame

VAT Vallecular aggregation time: EOA_frame-BPM_frame

EOA-HYB EOA-HYB interval: EOA_frame-HYB_frame

Time-to-LVC Time to most complete LVC: LVC_frame-HYB_frame

EOA-to-LVC EOA to LVC interval: LVC_frame-EOA_frame

HYB-to-UESO HYB to UESO interval: UESO_frame-HYB_frame

EOA-to-UESO EOA to UESO interval: UESO_frame-EOA_frame

LVCd LVC duration: LVCOff_frame-LVC_frame

UESOd UES opening duration: UESC_frame-UESO_frame

c) Bolus Location Measures

BLSO Bolus location at swallow onset: The location of the bolus head on the HYB_frame is recorded according to the following scale (Humbert et al., 2018):

1 = Oral cavity (anterior/superior to the ramus of the mandible)

2 = Between the ramus of the mandible and valleculae

3 = Within the valleculae; bounded by the leaf of the epiglottis

4 = Between the valleculae and pyriform sinus; including laryngeal surface of the epiglottis

5 = In pyriform sinuses; inferior to the arytenoids

6 = Within or inferior to the UES (esophagus)

d) Kinematic Measures

Note: All kinematic measures are scaled to the length of cervical spine scalar defined as the length of a line running between the anterior-inferior corners of the C2 and C4 vertebrae, and reported in %(C2-C4) units. (Molfenter & Steele, 2014).

UESMax UES maximum distention: the lateral diameter of the UES opening at its narrowest point on the UESMax_frame

PhAMPC Pharyngeal area at maximum constriction: the lateral area or any unobliterated space in the pharynx (containing air or bolus) on the MPC_frame. The boundaries of the pharynx are defined superiorly by the top of C2; posteriorly by the posterior pharyngeal wall; anteriorly by the base of tongue, pharyngeal surface of the epiglottis, and aryepiglottic folds; and inferiorly by the pit of the pyriform sinuses, superior to the UES.

Residue The lateral area of any residue visible within the boundaries of the pharynx on the SR_frame. This measure may be subdivided into residue within the valleculae, within the pyriform sinuses. and elsewhere in the pharynx.

References:

Humbert, I. A., Sunday, K. L., Karagiorgos, E., Vose, A. K., Gould, F., Greene, L., … & Rivet, A. (2018). Swallowing kinematic differences across frozen, mixed, and ultrathin liquid boluses in healthy adults: age, sex, and normal variability. Journal of Speech Language and Hearing Research, epub ahead of print. doi: 10.1044/2018_JSLHR-S-17-0417

Molfenter, S. M., & Steele, C. M. (2014). Use of an anatomical scalar to control for sex-based size differences in measures of hyoid excursion during swallowing. *Journal of Speech Language Hearing Research*, *57*(3), 768-778. https://doi.org/10.1044/2014_JSLHR-S-13-0152

Steele, C. M., Bayley, M. T., Bohn, M. K., Higgins, V., Peladeau-Pigeon, M., & Kulasingam, V. (2023). Reference Values for Videofluoroscopic Measures of Swallowing: An Update. *Journal of Speech Language Hearing Research*, *66*(10), 3804-3824. https://doi.org/10.1044/2023_JSLHR-23-00246
